# Integration of the Exogenous Tuning of Thraustochytrid Fermentation and Sulfur Polymerization of Single-Cell Oil for Developing Plant-like Oils

**DOI:** 10.3390/md20100655

**Published:** 2022-10-21

**Authors:** Adarsha Gupta, Max J. H. Worthington, Justin M. Chalker, Munish Puri

**Affiliations:** 1 Medical Biotechnology, Flinders Health and Medical Research Institute, College of Medicine and Public Health, Flinders University, Bedford Park, Adelaide, SA 5042, Australia; 2 Key Centre for Polymers and Colloids, School of Chemistry, The University of Sydney, Sydney, NSW 2006, Australia; 3 Institute for Nanoscale Science and Technology, College of Science and Engineering, Flinders University, Bedford Park, Adelaide, SA 5042, Australia

**Keywords:** cell factory, polyunsaturated fatty acids, omega-3 fatty acid, plant oils, fermentation, sulfur polymerization

## Abstract

In this study, we have demonstrated a bioprocessing approach encompassing the exogenous addition of low-molecular-weight compounds to tune the fatty acid (FA) profile in a novel thraustochytrid strain to produce desirable FAs. Maximum lipid recovery (38%, dry wt. biomass) was obtained at 1% Tween 80 and 0.25 mg/L of Vitamin B12. The transesterified lipid showed palmitic acid (C16, 35.7% TFA), stearic acid (C18, 2.1% TFA), and oleic acid (C18:1, 18.7% TFA) as the main components of total FAs, which are mainly present in plant oils. Strikingly, D-limonene addition in the fermentation medium repressed the production of polyunsaturated fatty acid (PUFAs). Sulfur-polymerization-guided lipid separation revealed the presence of saturated (SFAs, 53% TFA) and monounsaturated fatty acids (MUFAs, 46.6% TFA) in thraustochytrid oil that mimics plant-oil-like FA profiles. This work is industrially valuable and advocates the use of sulfur polymerization for preparation of plant-like oils through tuneable thraustochytrid lipids.

## 1. Introduction

Microbial oil (known as single-cell oil, SCO) has been regarded as a promising alternative for fossil fuels and plant-derived oils in recent years, due to its perceived sustainability, especially in the heterotrophic mode of cultivation. SCO from heterotrophic production has lower impacts on the environment due to less greenhouse gas emissions when compared with autotrophic production (that has longer production period) without compromising on the oil yields as shown by life cycle assessment study [1]. In addition, microbial platforms (e.g., thraustochytrids) could be developed as biorefinery setup for the complete utilisation of all bioproducts in order to realise the industrial production process of microalgal products for several applications, including food and human nutrition [2]. Moreover, microbial oil containing saturated and mono- and di-unsaturated fatty acids can mimic the oil composition of plant-derived oils, thus making it suitable for incorporation in food and food products with cost-effective production [3]. Furthermore, there is a growing awareness in humans with respect to dietary habits related to the consumption of vegetable oils and other related products [4]. Thus, the incorporation of thraustochytrid-derived oils could emerge as a new platform in the food industry since these oils have some distinct advantages for replacing plant-derived oils in current food products, including cocoa butter equivalents [5].

Thraustochytrids (especially *Schizochytrium* sp.), as heterotrophic marine microbes, are known for their rapid growth rate and lipid productivity (~50%, dry wt.) [6]. They are known as high producers of omega-3 fatty acids (DHA, docosahexaenoic acid) and saturated fatty acids (palmitic acid), and are amenable to fermentation optimisation with the use of several small molecules, including food-grade compounds, to tune fatty acid profiles in order to achieve a desirable oil composition [7]. In addition, their high biomass yield, which can only be achieved by heterotrophic production, makes them superior for industrial applications. Moreover, oils from this microbial group have attained GRAS (Generally recognised as safe) status for human consumption and have been deemed safe for infant nutrition [8,9]. Thraustochytrids accumulate large quantities of desirable saturated fatty acids and monounsaturated fatty acids in their cells [10]; thus, they can also be used as a source for producing oils mimicking plant-oil profiles.

Recently, the role of adaptative lab evolution as a non-genetic approach to improve lipid (especially DHA) accumulation [11] and introduction of chemical modulators in the fermentation medium have been proposed to improve lipid yields [7,12]. Low-molecular-weight compounds such as propyl gallate [7], butanol [13], ascorbic acid [14], naphthoxyacetic acid (BNOA) [12] and phytohormones, such as kinetin (KIN), jasmonic acid (JA) and gibberellic acid (GA) [15], and sesamol [16], when added to the fermentation medium, could enhance lipid production with reference to polyunsaturated fatty acids (DHA, etc.). Such studies have led to enhancements in lipid production, but so far have not investigated the effects on fatty acid content and PUFA downstream processing, which is essential to produce SCO mimicking plant-like oil profiles suitable for food and pharmaceutical applications.

Various methods, such as the use of organic solvents, ionic liquids, supercritical carbon dioxide (ScCO_2_) and urea complexation, among others, have been reported as chemical methods for the separation of PUFAs from microbial oils. However, each of these techniques have inherent limitations such as contaminants or residual non-edible substances that can limit their use in food and pharmaceuticals applications and ScCO_2_ extraction are expensive [17]. Thus, it was imperative to develop a downstream processing method to enrich oils with a desired degree of saturation or unsaturation. In this context, we recently demonstrated the use of elemental sulfur to separate saturated triglycerides and polyunsaturated triglycerides from thraustochytrid oil with the development of a polyunsaturated-triglyceride enriched sulfur polymer that has several applications [18]. The objective of the study was to tune the fatty acid profile of the thraustochytrid oil employing fermentation and subsequently use inverse vulcanization to separate saturated fatty acids from PUFAs (such as omega-3 fatty acids) so that the resultant oil can mimic the plant-oil-like profile.

In this study, an in-house thraustochytrid strain was subjected to different dosages of several exogenous compounds, such as vitamin B12, Tween 80, and D-limonene, at different concentrations in the fermentation medium for its ability to produce a plant-like fatty acid profile. The lipid profiles of oleaginous microorganisms were enhanced through bioprocessing intervention by adjusting culture conditions (which we term “tuning of fatty acid profiles”) to produce desirable fatty acid profiles that are analogous to terrestrial plant oils without genetic modification. Furthermore, the oil containing more saturated fatty acids (SFAs) and monosaturated (MUFAs) and less polyunsaturated fatty acid (PUFA) was extracted from the thraustochytrid biomass that was grown in an optimised fermentation medium (using the above-mentioned compounds), and extracted oil was subjected to further downstream processing using hydrogenation and a sulfur polymerization process. Heterogeneous catalytic hydrogenation has been used as a benchmark process for SFAs and MUFA production from PUFA-containing oil [18,19]. In addition, sulfur polymerization resulted in triglyceride-containing sulfur co-polymer because the sulfur reacts with the unsaturated triglycerides in the oil, with the chance of recovering SFA and MUFA containing oil from the polymer through ethanol extraction [18]. To the best of our knowledge, this is the first such study where upstream and downstream processing processes have been integrated to minimise challenges associated with biotechnological SCO production. A green, sustainable, and scalable method using sulfur polymerization was employed to separate the saturated fatty acids (SFAs) and monounsaturated fatty acids (MUFAs) and polyunsaturated fatty acids (PUFAs) from thraustochytrid oil. This mixture of SFAs and MUFAs in a thraustochytrid oil as extracted using ethanol from the sulfur polymer does mimic plant oils fatty acid profile that can be further used in food applications. The work here provided an effective strategy for enhancing desirable fatty acid production through tuning of *Schizochytrium* sp.

## 2. Results and Discussion

There has been increasing need for the development of alternative oils other than plant oils that can be used for human food and energy demand. Microbial oil can be developed as an alternative to plant oils since their fatty acid profile are similar, toxin free, and regarded as sustainable due to no requirement of large land use. In addition, the fatty acid profile could be tuned for specific applications (such as food) [5].

### 2.1. Exogenous Addition of Vitamin B12, Tween 80 and Limonene in Fermentation Medium

**Figure 1:**
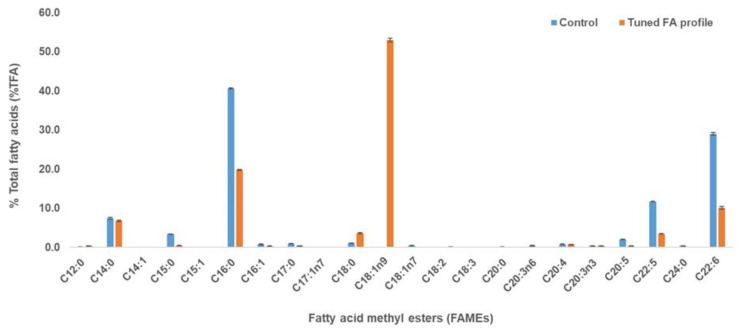
Fatty acid profiles of MASA#4 grown in glucose medium containing vitamin B12 (0.25 mg/L), Tween 80 (1%) and limonene (0.45%). The blue and orange bars show the FAMEs from the control and tuned FA profile, respectively. This oil was used in the downstream processing to separate the PUFAs from SFAs and MUFAs. Results are presented as mean ± standard deviation (SD) of triplicates.

In this study, heterotrophic thraustochytrid strain (MASA#4) was used with the aim to produce oil containing specific fatty acid profile close to that of plant oil (mainly containing SFAs and MUFAs). Different compounds, such as vitamin B12 (0.05, 0.1, 0.25, 0.5 and 1.0 mg/L), Tween 80 (0.5%, 1%, and 1.5%) and limonene (0.45%, 0.6% and 0.9%) were used to tune the fatty acid profile of MASA#4 strain with the objective of achieving the oil profile containing SFAs and MUFAs by reducing PUFAs (as a result of Tween 80 and limonene addition) and odd-chain fatty acid accumulation (with the use of Vitamin B12) (Figure 1). For all conditions, biomass was harvested on day 5 of the fermentation period. The optimised concentration of Vitamin B12, Tween 80 and limonene were determined as 0.25 mg/L, 1% and 0.45%, respectively, based on their fatty acid profiles (Supplementary Figures S1–S4). Different conditions resulted in various biomass and lipid production (the optimised concentration of Vitamin B12, Tween 80 and limonene showed biomass and lipid content of 12.7 g/L and 34.5%, 16.5 g/L and 52%, 10 g/L and 40.5%, respectively). Vitamin B12 resulted in the accumulation of odd-chain FAs (C15:0 and C17:0); thus, 0.25 mg/L was used as the optimum concentration in further experiments (Supplementary Figure S1). Similarly, Tween 80 at 0.5% resulted in low oleic acid content (10% of TFA) when compared with Tween 80 at 1% that accumulated higher oleic acid content (22% of TFA). More than 1% Tween 80 did not yield better results; thus, 1% of Tween 80 was determined as the optimum concentration (Supplementary Figure S2). When limonene was added at the onset of the fermentation medium, no biomass growth was observed; thus, limonene was added at 24 h and 48 h of fermentation duration and 48 h was determined as the optimum limonene concentration (Supplementary Figure S3). Maximum PUFA reduction was observed when 0.45% of limonene was used when compared with other limonene concentrations (Supplementary Figure S4).

When an optimised medium was used with optimised concentrations of Vitamin B12, Tween 80 and D-limonene, the major fatty acids in the control and tuned fatty acid profile were C14:0 (7.4% and 6.8% of TFA), C15:0 (3.4% and 0.4% of TFA), C16:0 (40.6% and 19.8% TFA), C18:0 (1.2% and 3.6% of TFA), C18:1n9 (0% and 53.0% of TFA), C20:5 (2.1% and 0.3% of TFA), C22:5 (11.7% and 3.5% of TFA) and C22:6 (29.0% and 10.1% of TFA), respectively (Figure 1). The tuned FA profile of thraustochytrid-derived oil resembled a plant-like oil profile showing the accumulation of SFA and MUFA with reduced PUFAs when compared with control, in which PUFA accumulation is high. Vitamin B12 (0.25 mg/L) in the medium reduced the odd chain fatty acid (C15:0) from 3.4 to 0.4% of TFA (Figure 1). It has been reported that vitamin B12 can activate methylmalonyl-CoA mutase in thraustochytrid cells converting the propionic acid into succinic acid, thus resulting in the unavailability of odd-chain FA precursor [20]. Furthermore, the addition of Tween-80 (1%) acted as a precursor for oleic acid synthesis in the fatty acid profile of thraustochytrid strain [21].

Similarly, the addition of D-limonene (0.45% added at 48 h of optimum fermentation period) resulted in the decrease in PUFAs, including DHA, as it can inhibit desaturase and elongase enzymes. It has been reported that citrus oil (containing limonene content) resulted in PUFA decreases in *Yarrowia lipolytica* [22]. In this study, saturated FAs, such as (C14:0, C16:0, and C18:0) and monounsaturated FA (C18:1n9) and PUFAs were maintained, improved and reduced, respectively, compared with the control (Figure 1). This was in agreement with a previous study of *Y. lipolytica*, in which C12 and C14 improved due to the inhibition of acyl-SCoA elongase that catalyses the following reaction: C12:0 --> C14:0 --> C16:0 [22]. In addition, C18:0 and C18:1 improved at low concentration of limonene, similar to this current study. Similar findings have been reported when essential oil containing limonene was used to tailor the fatty acid profile in oleaginous yeasts resulting in higher saturated fatty acids [23].

### 2.2. Downstream Processing: Hydrogenation of Thraustochytrid Oil

When optimised medium was used, 10 g/L of freeze-dried biomass and ~30% lipid content (dry wt. basis) was obtained. Low biomass could be attributed to the addition of limonene in the fermentation medium [22]. For downstream processing, 10 g of freeze-dried biomass was incubated with solvent mixture (chloroform: methanol; 2:1). The mixture was used for oil extraction using homogenization and the subsequent solvent layer was taken and filtered before evaporating the solvent to obtain oil sample. For the hydrogenation of the oil, the average yield of isolated hydrogenated oil from three trials was 70% yield by mass.

Vegetable oils are mainly composed of oleic acid (C18:1), linoleic acid (C18:2), stearic acid (C18:0) and palmitic acid (C16:0) [24]. Specifically, the main fatty acids (in % wt.) present in canola oil, olive oil and sunflower oil are oleic acid (78.7%, 77.7%, 37.3%), linoleic acid (14.2%, 8.9%, 50%), palmitic acid (4%, 9.9%, 0.06%) and stearic acid (1.8%, 2.3%, 5.4%), respectively [25]. It is well known that highly unsaturated oils are subjected to selective heterogeneous catalytic hydrogenation for monoene production that can be used in the development of several products, such as bio-lubricants and biofuels [19].

### 2.3. Copolymerization of Thraustochytrid Oil with Sulfur

**Figure 2:**
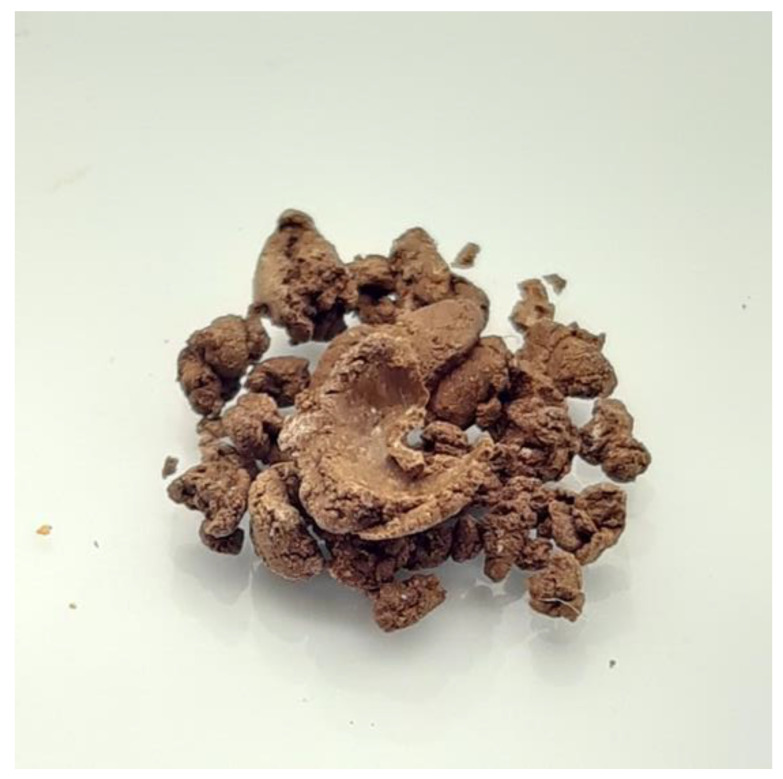
The reaction of sulfur and thraustochytrid oil resulted in thraustochytrid oil polymer exhibiting brown sticky mixture and thickened on cooling.

The oil obtained after homogenization-assisted solvent extraction was used for copolymerization with sulfur. This copolymerization of the oil and sulfur resulted in a brown sticky solid, which thickened on cooling (Figure 2). Of a starting mass of 559 mg of oil and 505 mg sulfur, 330.4 mg unreacted, and concentrated oil was extracted with ethanol.

**Figure 3:**
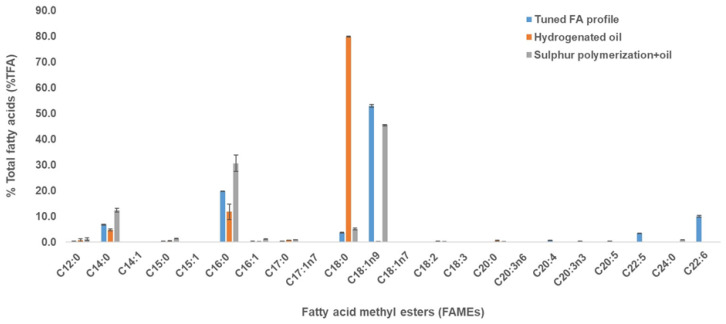
Fatty acid profile of the thraustochytrid oil obtained after hydrogenation and sulfur polymerization process. The blue, orange and grey bars show the FAMEs from the tuned FA profile, hydrogenated oil, and sulfur-polymerized oil, respectively. No polyunsaturated fatty acids (PUFAs) were detected in the FA profile of the sulfur-polymerized oil sample (grey bars). Results are presented as mean ± standard deviation (SD) of triplicates.

**Figure 4:**
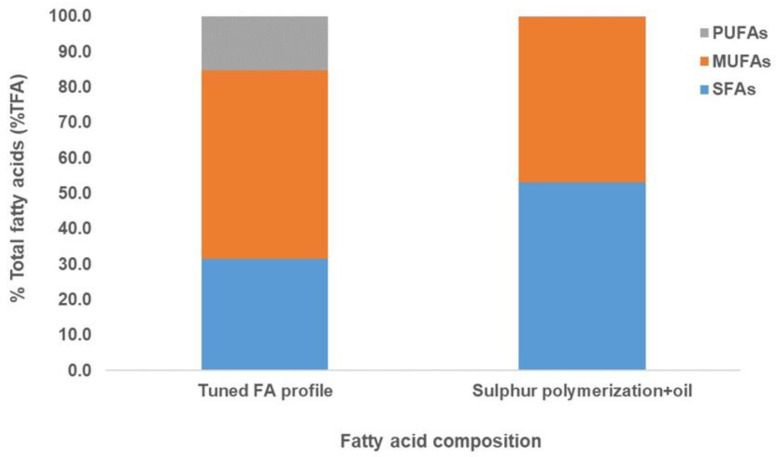
Fatty acid profile of the thraustochytrid oil obtained after tuning of the fermentation and sulfur polymerization process. The blue, orange and grey bars show SFAs, MUFAs and PUFAs, respectively. No polyunsaturated fatty acids (PUFAs) were detected in the FA profile of the sulfur-polymerized oil sample (grey bars).

The solid polymer in Figure 2 was formed primarily by the reaction of elemental sulfur with the PUFAs in the thraustochytrid oil (the solid yield was around 50%). Unreacted oil was extracted with ethanol, as described above. The extracted oil was primarily saturated and monounsaturated oils (Figure 3 and Figure 4) exhibiting SFAs and MUFAs (with no PUFAs). The remaining solid, however, can also be considered a value-added product as these sulfur rich polymers have been used in a number of applications, including environmental remediation [25,26], cathode materials for Li-S batteries [27], precision fertilisers [28] and composite materials for construction [29,30,31].

### 2.4. Fatty Acid Profile of the Oil Samples after Hydrogenation and Sulfur Polymerization

Almost 30% lipid was obtained by homogenization-assisted solvent extraction. These lipid samples were used for the hydrogenation and sulfur polymerization process.

In hydrogenation processes, the use of specific catalysts (Ni and Pd) exhibits low selectivity towards monoenes/dienes, resulting in the formation of saturated FAs (SFAs) [19]. This was in agreement with this study as the hydrogenation of thraustochytrid oil (containing high oleic acid and other PUFAs) produced a mixture that contain high stearic acid (Figure 3).

The sulfur polymerization process resulted in a sulfur polymer from which unreacted oils could be extracted with ethanol. These unreacted oils were primarily C16 and C18:1n9 and not PUFAs (Figure 3). In both processes, the oil sample showed no detection of polyunsaturated fatty acids (PUFAs), and saturated fatty acids (SFAs) were maintained in the fatty acid profile when compared with the tuned FA profile (Figure 3 and Figure 4). Thus, these results showed that both the hydrogenation and sulfur polymerization processes could separate PUFAs from the oil sample, concentrating mainly oleic acid and palmitic acid, thus mimicking plant-like oils (Figure 3 and Figure 4).

In this study, after the sulfur polymerization process, PUFAs in the thraustochytrid oil reacted with sulfur and formed a sulfur polymer. The unreacted SFAs and MUFAs were separated from the mixture to form an oil fraction resembling a plant-like oil profile. Thus, this is a novel method of separating PUFAs (forming a co-polymer with sulfur) and the SFAs-MUFAs fraction from thraustochytrid oil. Earlier, several downstream processing of PUFAs have been reported using solvents, including the use of cell disruption methods and other emerging technologies that include the use of supercritical carbon dioxide, dimethyl carbonate, and eutectic solvents along with winterization, molecular distillation, enzymatic purification, low-temperature crystallization, purification by urea inclusion, and chromatographic separation [17,32,33].

### 2.5. Characterisation of the Thraustochytrid Oil and the Oil-Sulfur Polymer

The fatty acid profile of the oil after hydrogenation showed no MUFAs and PUFAs (Figure 3), which was consistent with the NMR analysis of the oil (in methanol-d4) that showed no presence of alkenes (Supplementary Figure S5a). Similarly, after sulfur polymerization, the FA profile showed only SFAs and MUFAs with no PUFAs (Figure 3 and Figure 4), due to the reaction of sulfur with the alkenes of the PUFAs to form an insoluble copolymer easily separated from other unreacted saturated and MUFAs. This was also validated by ^1^H NMR spectroscopy, when the oil was dissolved in CDCl3 before sulfur polymerization, an average of 4.99 alkenes were found to be present per triglyceride molecule, which decreased to 1.19 alkenes per triglyceride after the sulfur polymerization, around 76% less (Supplementary Figure S5b,c). Nuclear Magnetic Resonance (NMR) spectroscopy is a well-recognised technique to check the identity and structure of chemical compounds. NMR has been used to characterise several vegetable oils [34] and it provides unique approaches for the analysis of existing saturation/unsaturation in thraustochytrid oil with the detection of alkenes molecules.

The thraustochytrid oil polymer was characterised to compare its composition with the resultant polymers produced from the sulfur polymerization with vegetable oils [27,35]. The thraustochytrid oil polymer was malleable similar to the soft and rubbery nature of the sulfur–vegetable oils polymer previously reported [25,35]. The SEM, EDS, STA, and XRD analysis showed considerable amounts of sulfur (Supplementary Figures S6–S8). In SEM analysis, smooth sulfur rich polymer was observed with small regions of crystalline sulfur and EDX elemental map showed the distribution of sulfur, carbon and oxygen on the surface (Supplementary Figure S6a–c). The STA analysis demonstrated that roughly half of the mass of the resultant polymer was unreacted sulfur (Supplementary Figure S7a), which is in agreement with previous observation that the oil and sulfur polymer contain significant quantities of free sulfur [25]. Furthermore, XRD analysis revealed that the spectrum appears to be identical for crystalline S_8_, confirming that the polymer co-product can be co-isolated with unreacted sulfur (Supplementary Figure S8).

The present protocol has the potential for scaling-up from laboratory scale to an industrial scale. This study is an important step forward in the sustainable production of single-cell oils that mimic plant-oil-like fatty acid composition by following precision fermentation approach, thus supporting a paradigm shift towards risk-reduced food manufacturing.

In summary, a novel process was developed to achieve the separation of microbial oil containing SFAs and MUFAs (mainly C16:0 and C18:1n9) from PUFAs. Thus, thraustochytrid oil containing desirable FA profile was obtained with no PUFAs. This oil, without PUFAs, mimics plant-like oil composition that has the potential to replace plant oils for its incorporation in food products. Furthermore, the separated PUFAs were incorporated in a sulfur polymer that can be a useful material for various applications, including agricultural fertilizers [28], insulation [30], oil spill [26], heavy metal [35] and PFOA remediation [36]. This study warrants the development of an optimised process at a larger scale.

## 3. Materials and Methods

### 3.1. Medium Components and Chemicals

All medium components were procured from Sigma-Aldrich, Australia unless otherwise stated. Instant ocean sea salts (Blacksburg, VA, USA) were used to prepare artificial seawater (ASW). The solvents used in lipid extraction were purchased from Merck Chemicals (Sydney, NSW, Australia) and were of either analytical or molecular grade.

### 3.2. Thraustochytrid Strain and Inoculum Preparation

An in-house thraustochytrid strain, isolated from South Australian marine waters, was used in this study. The isolate was identified based on 18S rRNA gene sequences and found to be closely related to other 18S rRNA sequences from thraustochytrid strains available in the GenBank database. As a result, this strain was found to be closely related to *Schizochytrium* sp.; thus, it has been designated as *Schizochytrium* sp. MASA#4. The strain was sub-cultured fortnightly in the agar medium (glucose 10 g/L, yeast extract 1 g/L, peptone 1 g/L, agar 10 g/L and artificial seawater, ASW 50%) and incubated at 25 °C. Inoculum was prepared in medium containing above-mentioned medium (without agar) and incubated for 48 h at 25 °C at 150 rpm. All the experiments were conducted in shake flasks (50 mL medium in 250 mL flasks) and incubated at 25 °C, 150 rpm for five days, unless mentioned otherwise.

### 3.3. Exogenous Supplementation of Chemical Modulators in the Fermentation Medium

In this study, different compounds, such as vitamin B12 (0.05, 0.1, 0.25, 0.5 and 1.0 mg/L), Tween 80 (0.5%, 1%, and 1.5%) and limonene (0.45%, 0.6% and 0.9%) were optimised to tune the fatty acid profile of MASA#4 strain. This strain was grown in a fermentation medium containing glucose-5%, yeast extract-0.4%, peptone-0.04%, monosodium glutamate (2%), magnesium sulphate (1%), vitamin B12–0.25 to 1.0 mg/L, Tween 80–0.5 to 2% and incubated at 25 °C at 150 rpm. D-Limonene (0.45–0.9%) was used at different concentrations in the fermentation medium at different time duration (in hours, h).

### 3.4. Biomass Production

The *Schizochytrium* strain was grown in a 5 L flask to produce the biomass required for this study. Inoculum (10%) was used in the production medium containing glucose 50 (g/L), yeast extract (4 g/L), peptone (0.04 g/L), MgSO_4_ (10 g/L), and monosodium glutamate (20 g/L), vitamin B12-0.25 mg/L, Tween 80-1% and limonene (0.45%, added at 48 h of incubations) and cultivated at 25 °C, 150 rpm for 5 days. The culture medium was harvested after 5 days and subjected to freeze drying to obtain dried biomass (Virtis freeze dryer, Gardiner, NY, USA).

### 3.5. Lipid Extraction, FAMEs Preparation and GC Analysis

For lipid extraction, freeze-dried biomass (2.0 g) was subjected to homogenization (Ultra-Turrax^®^ T 25, IKA, Staufen, Germany) (220 V, 50/60 Hz, 600 W) in a chloroform: methanol mixture (2:1, *v*/*v*) at 20,000 rpm for 2 min. The homogenization was repeated three times and all the extracts were combined and filtered through a 0.22 µm filter. The solvent was evaporated using a rotary evaporator (bath temperature at 50 °C) (Buchi, Flawil, Switzerland) and lipid weight was determined gravimetrically [6].

Lipids were transesterified and fatty acid methyl esters (FAMEs) were analysed using a previously described method [6]. FAME analysis was performed on a Shimadzu Gas chromatography (GC, 2090 N) (Kyoto, Japan) equipped with flame ionisation detector (FID) and connected to a BID 2030 unit using FAMEWAX column (30 m × 0.32 mm ID (inner diameter)). The inlet was held at 25 °C with a constant column flow rate at 5 mL/min with split injection (1/150). The oven program was held at 150 °C (5 min hold), ramped to 250 °C at a rate of 10 °C per min. and held at 250 °C for 1 min. Fatty acid esters were identified by comparison of peak areas of FAMEs standards (Sigma Aldrich, Castle Hill, NSW, Australia, CRM47885).

### 3.6. Hydrogenation of Thraustochytrid Oil

The hydrogenation process was conducted with modifications [19]. Thraustochytrid oil (500 mg) was dissolved in 15 mL ethanol in a 50 mL round bottom flask. Subsequently, 20 mg palladium on carbon (10 wt. % loading) was added, the flask was sealed with a septum and sparged with hydrogen gas for 20 min. The mixture was left to react at room temperature with magnetic stirring for 3 days. The mixture was filtered over celite (SiO_2_) under vacuum to remove Pd/C and concentrated under reduced pressure to isolate the oil.

### 3.7. Copolymerization of Thraustochytrid Oil with Sulfur

Thraustochytrid oil (559 mg) was dissolved in warm ethanol to transfer to a 100 mL round bottom flask and dried under vacuum. The mixture was heated to 170 °C on an aluminium heating block with magnetic stirring. After 5 min of stirring at that temperature, 505 mg elemental sulfur (elemental sulfur: oil ratio at around 1:1) was added gradually to the oil over a period of 4 min [26]. A heat gun was used to melt sulfur that adhered to the glass walls of the flask. After stirring for 1 h 45 min at 170 °C, the flask was removed from the hot block and the (still liquid) mixture was left to cool. The oil thickened to a sticky substance on cooling, presenting as a mixture of solid sulfur, a co-polymer made from the reaction of sulfur with unsaturated triglycerides, and unreacted oil.

### 3.8. Analysis of Thraustochytrid Oil Recovered after Hydrogenation or after Copolymerization with Sulfur

After hydrogenation, 10 mg of the oil sample was directly used for transesterification and GC analysis following the FAME analysis, as mentioned above. However, after the sulfur polymerization process, the polymer containing oil was subjected to ethanol extraction (20 mL) at 50 °C for 2 h. The ethanol in the solvent extract was evaporated using a rotary evaporator at 50 °C (R-300, Buchi, Flawil, Switzerland) and lipid weight was determined gravimetrically; 10 mg of this dried lipid was used for transesterification to prepare FAMEs and subsequent GC analysis as described in Section 3.5.

The thraustochytrid oil after hydrogenation was characterised using NMR spectroscopy. Furthermore, oil polymer after sulfur polymerization was characterised using several techniques such as nuclear magnetic resonance (NMR) spectroscopy, scanning electron microscopy (SEM), energy-dispersive X-ray spectroscopy (EDS), thermogravimetric analysis (TGA), dynamic scanning calorimetry (DSC), and X-ray diffraction (XRD). The methodology and results are provided as Supplementary information.

## Data Availability

Data are available from the corresponding author.

## Supplementary Materials

The following supporting information can be downloaded at: https://www.mdpi.com/article/10.3390/md20100655/s1. The MS Word file contains the experimental data shown in Figures S1 to S8. Figure S1: Effect of different Vitamin B12 concentrations on the FA profile of MASA#4 strain. Figure S2: Effect of different Tween 80 concentrations on the FA profile of MASA#4 strain. Figure S3: Effect of time-dependent addition of D-limonene on the FA profile of MASA#4 strain. Figure S4: Effect of different D-limonene concentrations on the FA profile of MASA#4 strain. Figure S5a: ^1^H NMR spectrum of thraustochytrid oil in methanol-d4 after hydrogenation. Figure S5b: ^1^H NMR spectrum of soluble fraction of thraustochytrid oil in CDCl3. Figure S5c: ^1^H NMR spectrum of soluble fraction of thraustochytrid oil in CDCl3 after inverse vulcanisation. Figure S6a: SEM micrograph and corresponding EDX elemental map of thraustochytrid oil polymer. Figure S6b: SEM micrograph and corresponding EDX elemental map of thraustochytrid oil polymer. Figure S6c: Elemental map of thraustochytrid oil polymer by EDX. Figure S7a: STA analysis the polymer product of inverse vulcanisation of thraustochytrid oil. Figure S7b: DSC trace of thraustochytrid oil polymer. Figure S7c: DSC trace of thraustochytrid oil polymer, focused only on the baseline shift in the cooling and heating phases characteristic of a glass transition. Figure S7d: ^1^H NMR spectrum of thraustochytrid oil polymer in pyridine-d5. Figure S8: XRD spectrum of thraustochytrid oil polymer.
