# Supplementary material for: Integration of the Exogenous Tuning of Thraustochytrid Fermentation and Sulfur Polymerization of Single-Cell Oil for Developing Plant-like Oils
Source: Mar Drugs. 2022 Oct 21;20(10):655. doi: 10.3390/md20100655 (PMC9604933; doi:10.3390/md20100655)
Supplement: Supplementary file 1 [file marinedrugs-20-00655-s001.zip › marinedrugs-1913539-supplementary.pdf]

Integration of the Exogenous Tuning of Thraustochytrid Fermentation and Sulfur Polymerization of Single-Cell Oil for Developing Plant-like Oils

Adarsha Gupta[1], Max J. H. Worthington[2,3], Justin M. Chalker[3] and Munish Puri[1],*

[1] Medical Biotechnology, College of Medicine and Public Health, and Flinders Health and Medical Research Institute, Flinders University, Bedford Park, Adelaide, SA 5042, Australia

[2] Key Centre for Polymers and Colloids, School of Chemistry, The University of Sydney, Sydney, NSW 2006, Australia

[3] Institute for Nanoscale Science and Technology, College of Science and Engineering, Flinders University, Bedford Park, Adelaide, SA 5042, Australia

* Correspondence: munish.puri@flinders.edu.au; Tel.: +61-8-72218573

Supplementary figures captions (Fermentation optimization)

Figure S1: Effect of different Vitamin B12 concentrations on the FA profile of MASA#4 strain

Figure S2: Effect of different Tween 80 concentrations on the FA profile of MASA#4 strain

Figure S3: Effect of time-dependent addition of D-limonene on the FA profile of MASA#4 strain

Figure S4: Effect of different D-limonene concentrations on the FA profile of MASA#4 strain

Figure S1

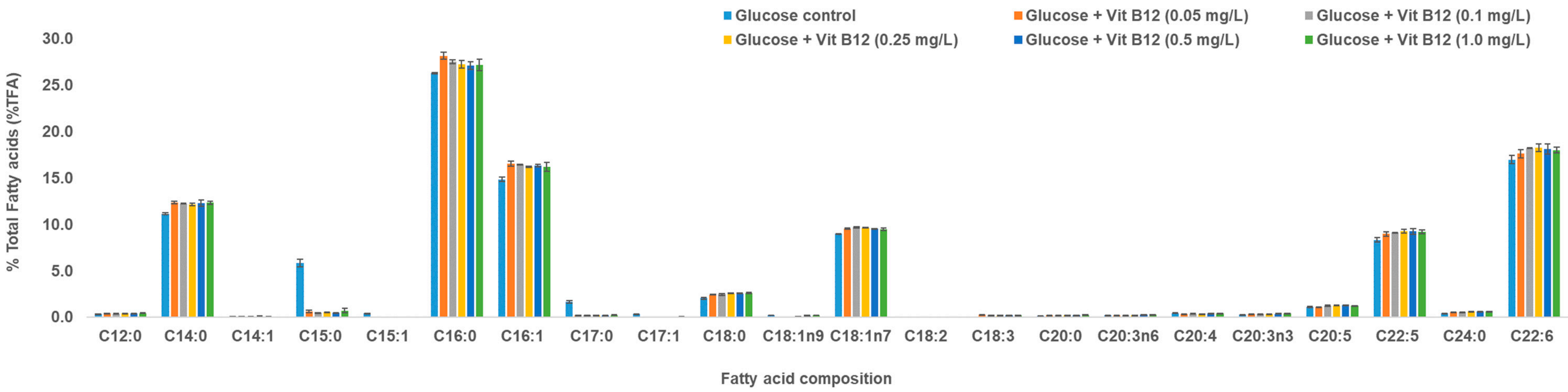

Figure S1: Effect of different Vitamin B12 concentrations on the FA profile of MASA#4 strain

Figure S2

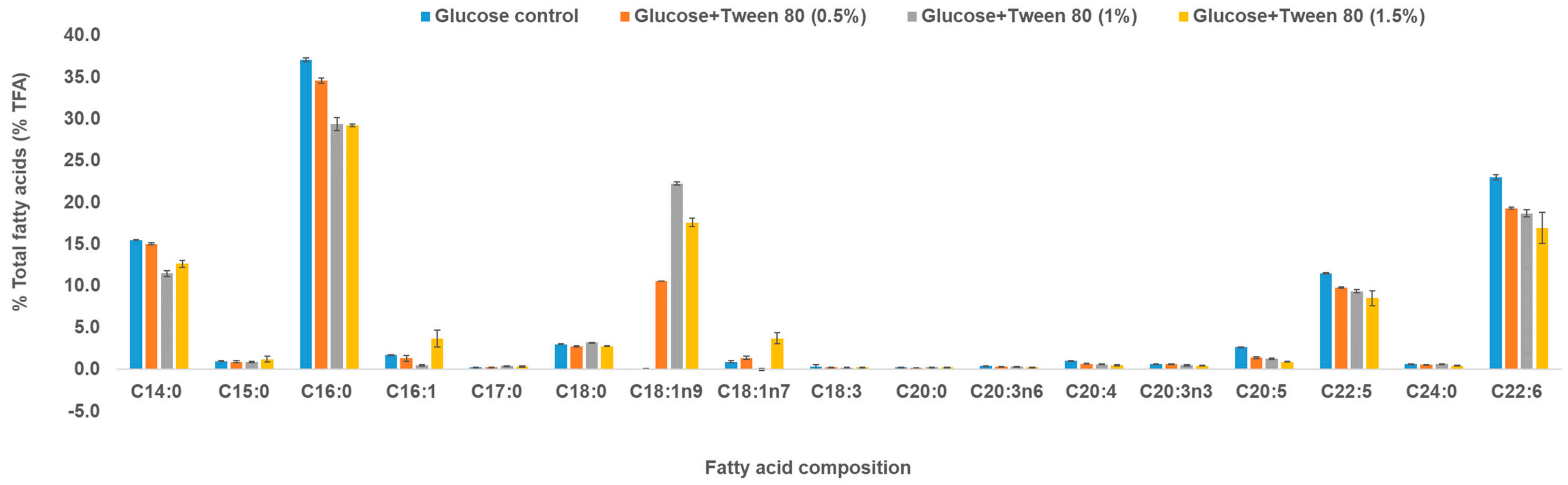

Figure S2: Effect of different Tween 80 concentrations on the FA profile of MASA#4 strain

Figure S3

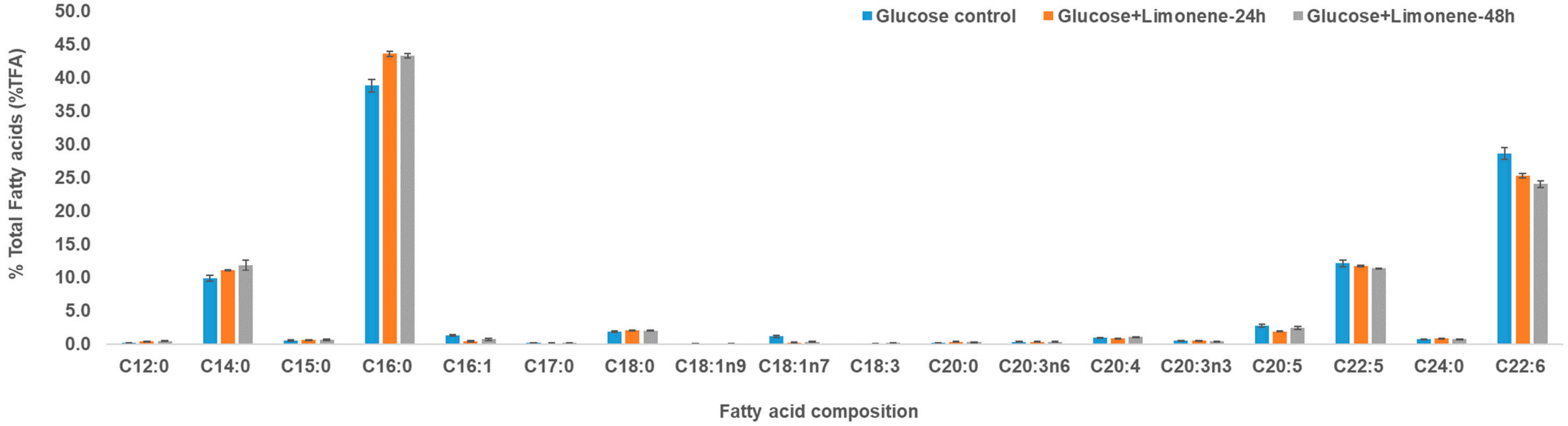

Figure S3: Effect of time-dependent addition of D-limonene on the FA profile of MASA#4 strain

Figure S4

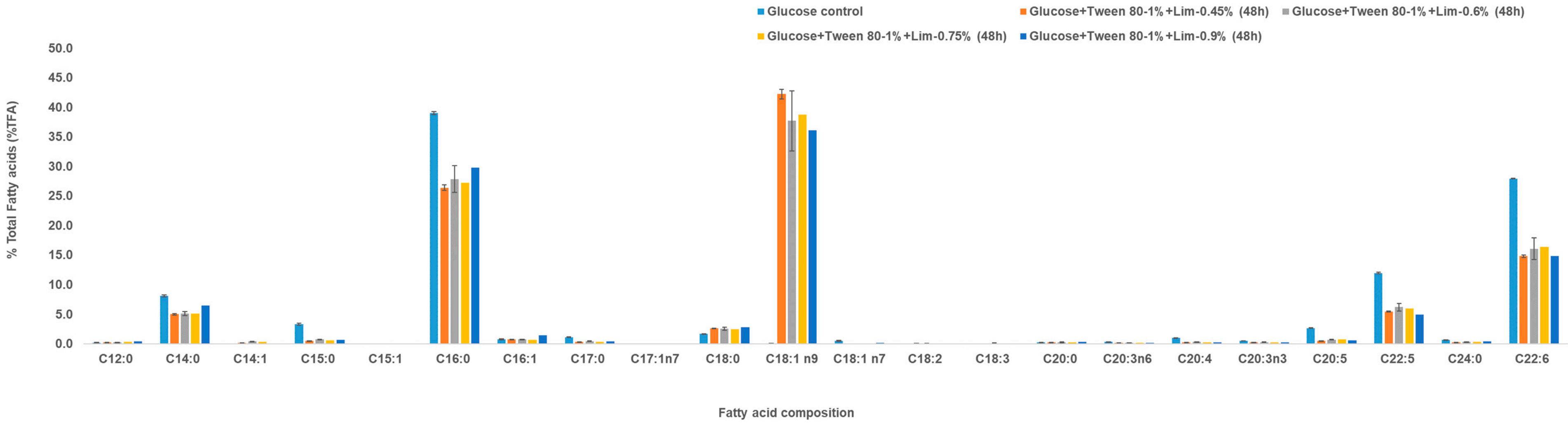

Figure S4: Effect of different D-limonene concentrations on the FA profile of MASA#4 strain

General experimental considerations (Characterisation of the sulfur polymer)

NMR Spectroscopy: Proton nuclear magnetic resonance (^{1}H NMR) (Bruker) spectra were recorded on a 600 MHz spectrometer or 400 MHz spectrometer where noted. All chemical shifts are quoted on the δ scale in ppm using residual solvent as the internal standard (^{1}H NMR: $CDCl_3$ δ = 7.26, methanol-d_4 δ = 4.87 and pyridine-d_5 δ = 8.74).

SEM and EDS: Scanning Electron Microscopy (SEM) images were obtained using an FEI F50 Inspect system, while corresponding EDS spectra were obtained using an EDAX Octane Pro detector.

Thermogravimetric Analysis (TGA): Simultaneous Thermal Analysis (STA) was carried out on a Perkin Elmer STA8000 simultaneous thermal analyzer. A sample size between 10 and 15 mg was used in each run. The furnace was purged at 20 mL/min with nitrogen, and equilibrated for 1 minute at 30 ºC before each run. Heating was carried out up to 700 ºC using a 20 ºC/min heating rate. The temperature was held isothermally at 700 ºC at the end of each experiment to oxidize remaining organic matter.

Differential Scanning Calorimetry (DSC): In order to determine glass transition temperatures, thermal analysis was performed on a Perkin Elmer DSC8000. A sample size between 5 and 10 mg was used in each run. The furnace was purged at 20 mL/min with nitrogen, and equilibrated for 1 minute at 30 ºC before each run. Heating was carried out up to 140 ºC using a 10 ºC/min heating rate, followed by a cooling step down to -60 °C at the same rate. Heating and cooling from -60 °C to 140 °C and back was repeated twice more to monitor any changes in the DSC profile over multiple cycles.

X-ray Diffraction: Powder X-ray diffraction (XRD) patterns were recorded on a Bruker D8 Advance Eco diffractometer (Bragg-Brentano geometry) using Co-Kα radiation (λ = 1.78897Å). The Bragg angle (2θ) was varied from 15° to 90° with a step size of 0.019°, measurement time of 0.45 s per step and sample rotation at 15 rpm. The XRD patterns were collected on a silicon low background sample holder, where powder samples were deposited onto the surface of the holder and spread evenly using a drop of acetone.

Characterisation of Thraustochytrid Oil after Hydrogenation and Sulfur Polymerization

NMR Spectroscopy

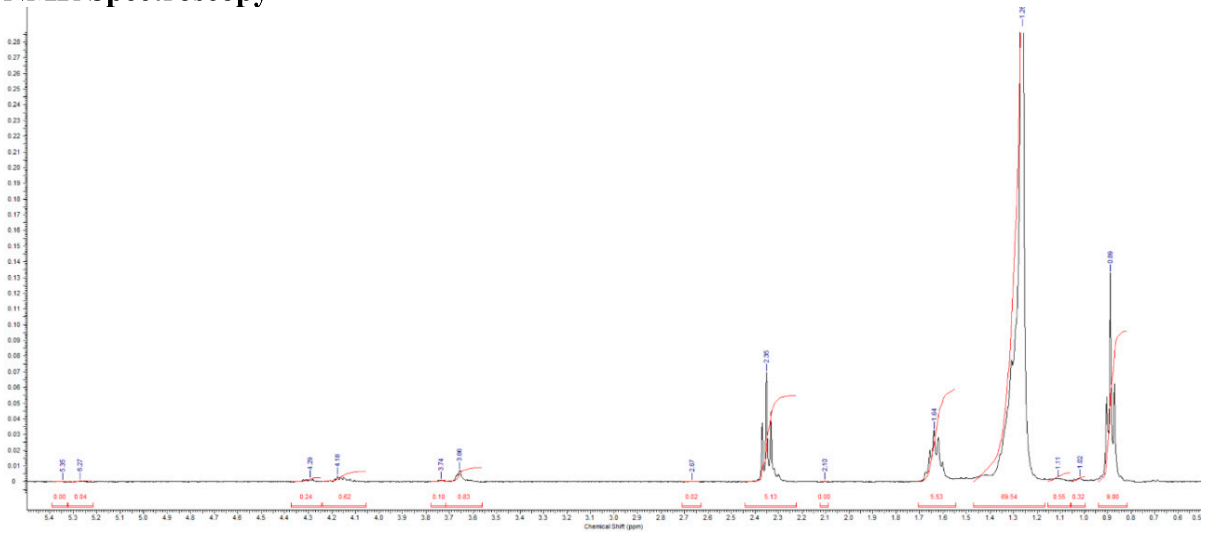

Figure S5a | ^{1}H NMR spectrum of thraustochytrid oil in methanol-d4 after hydrogenation acquired on a 400 MHz spectrometer. The spectrum indicates total conversion with an average of zero alkenes remaining (shown as 0.00 alkene protons, δ = 5.35) per triglyceride molecule. Methyl end-group protons were used as a reference (9 per molecule, δ = 0.92 ppm).

NMR Spectroscopy

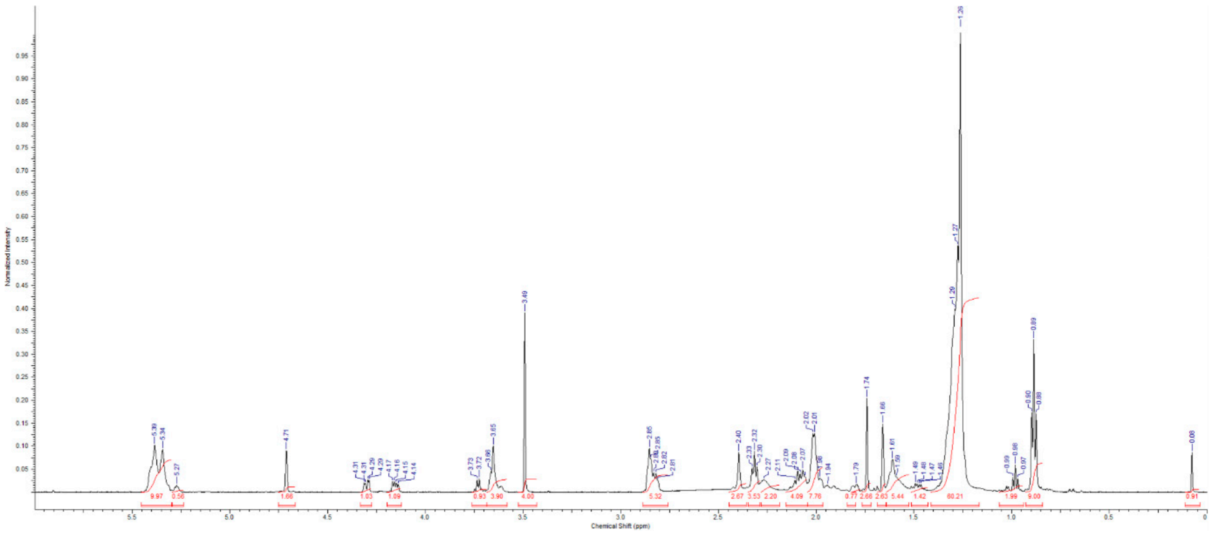

Figure S5b | ^{1}H NMR spectrum of soluble fraction of thraustochytrid oil in $CDCl_3$ acquired on a 400 MHz spectrometer. The spectrum indicates an average of 4.99 alkenes (shown as 9.97 alkene protons, sum of δ = 5.34 and 5.39 ppm) per triglyceride molecule, methyl end-group protons were used as a reference (9 per molecule, δ = 0.89 ppm).

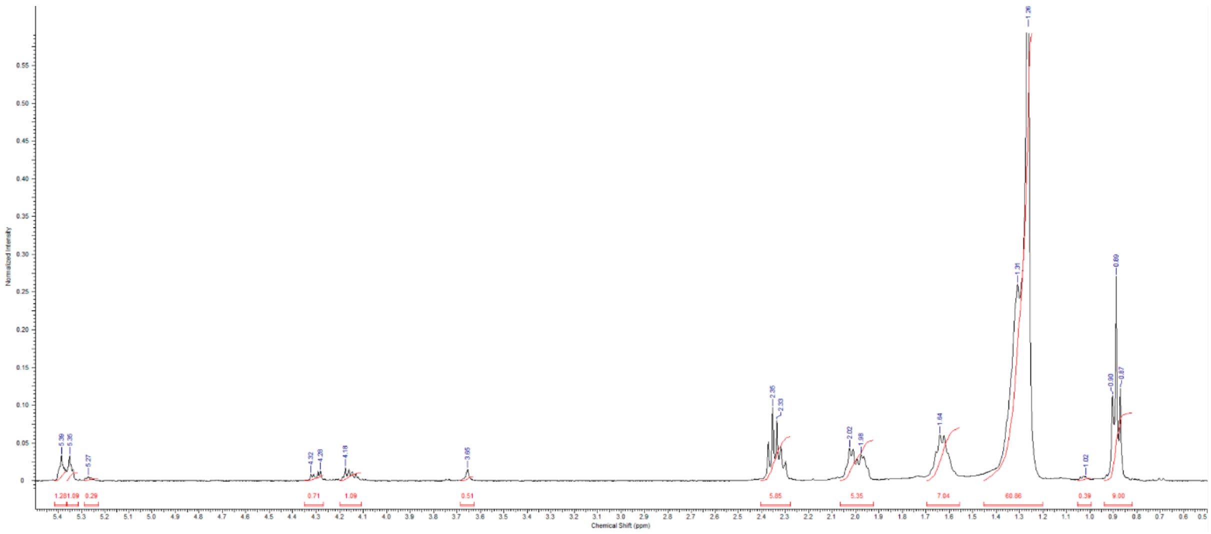

Figure S5c | ^{1}H NMR spectrum of soluble fraction of thraustochytrid oil in $CDCl_3$ after inverse vulcanisation acquired on a 400 MHz spectrometer. The spectrum indicates an average of 1.19 alkenes (shown as 2.37 alkene protons, sum of δ = 5.35 and 5.39 ppm) per triglyceride molecule, methyl end-group protons were used as a reference (9 per molecule, δ = 0.89 ppm).

Scanning Electron Microscopy and Electron Dispersive X-Ray Spectroscopy (SEM and EDS)

Thraustochytrid oil Polymer

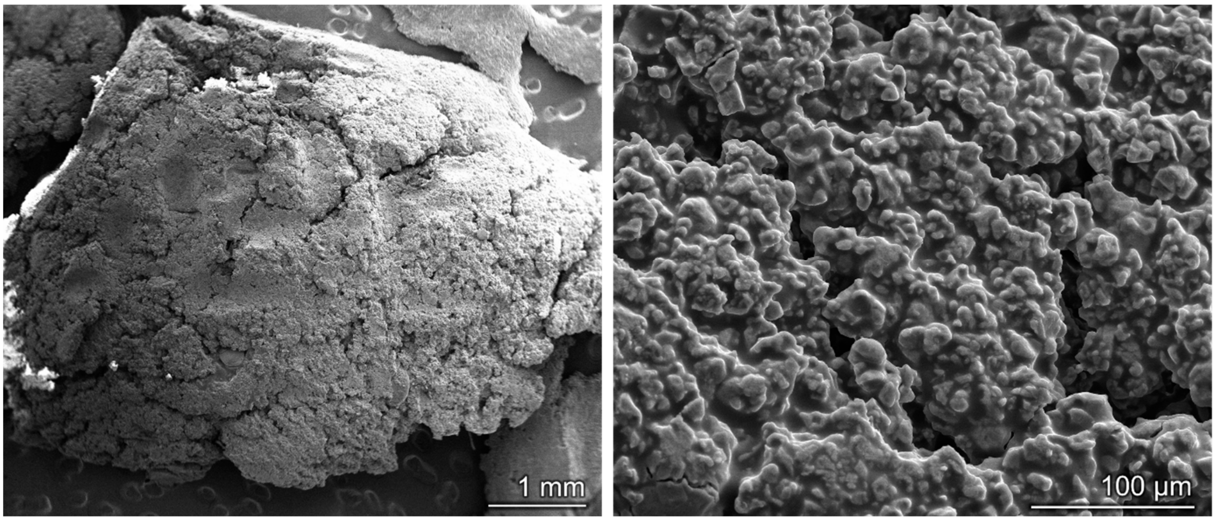

Figure S6a | SEM micrograph and corresponding EDX elemental map of thraustochytrid oil polymer. The map has been brightness-adjusted for visual clarity.

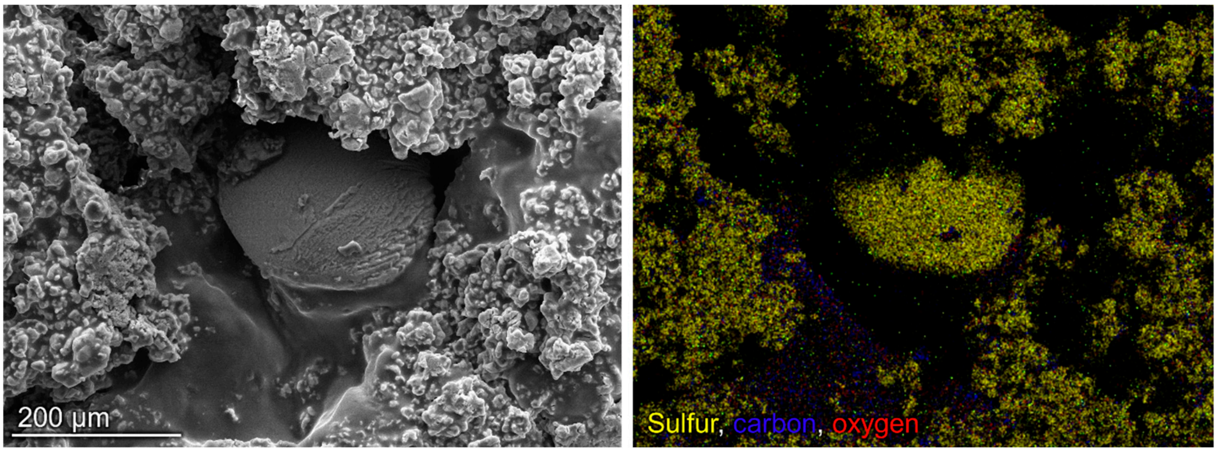

Figure S6b | SEM micrograph and corresponding EDX elemental map of thraustochytrid oil polymer. The map has been brightness-adjusted for visual clarity.

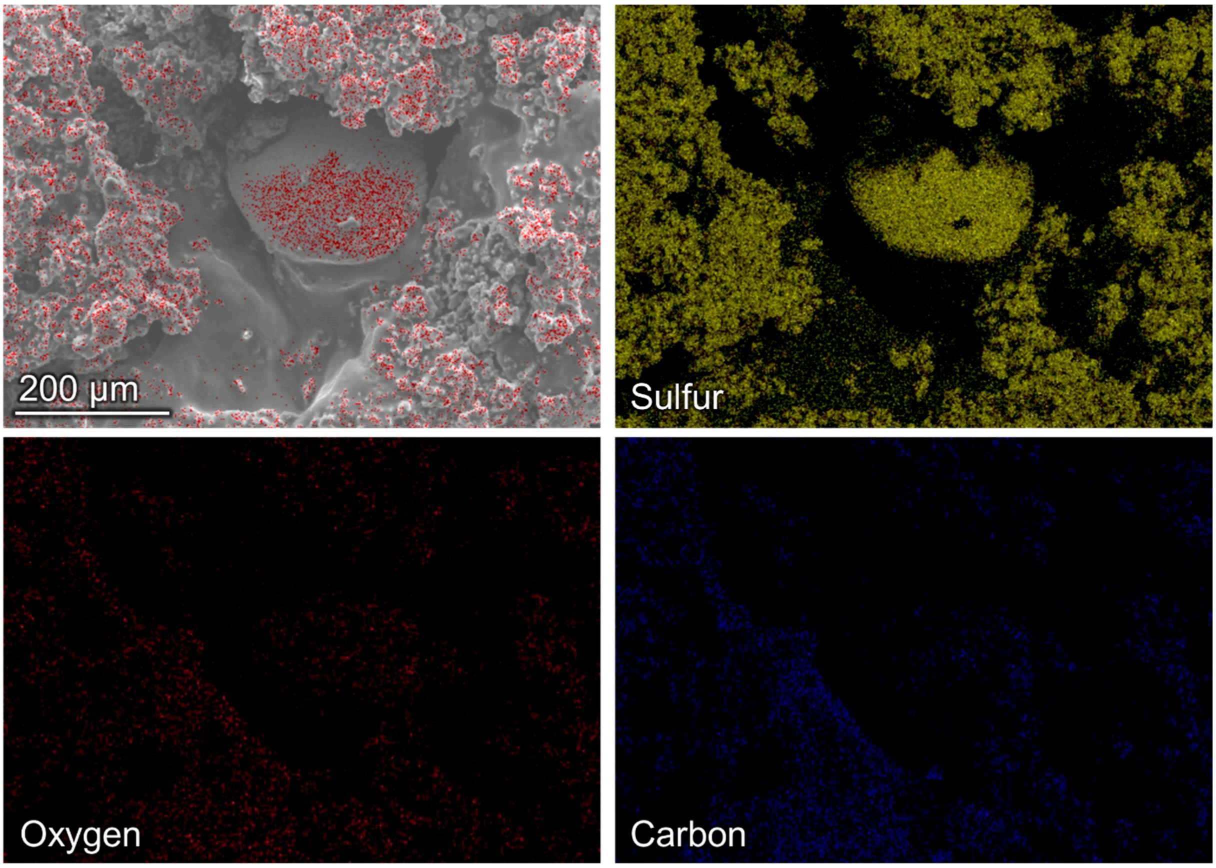

Figure S6c | Elemental map of thraustochytrid oil polymer by EDX. Top left maps where the signal was concentrated on the sample. Elemental images have had contrast and brightness adjusted for visual clarity.

Simultaneous Thermal Analysis (STA)

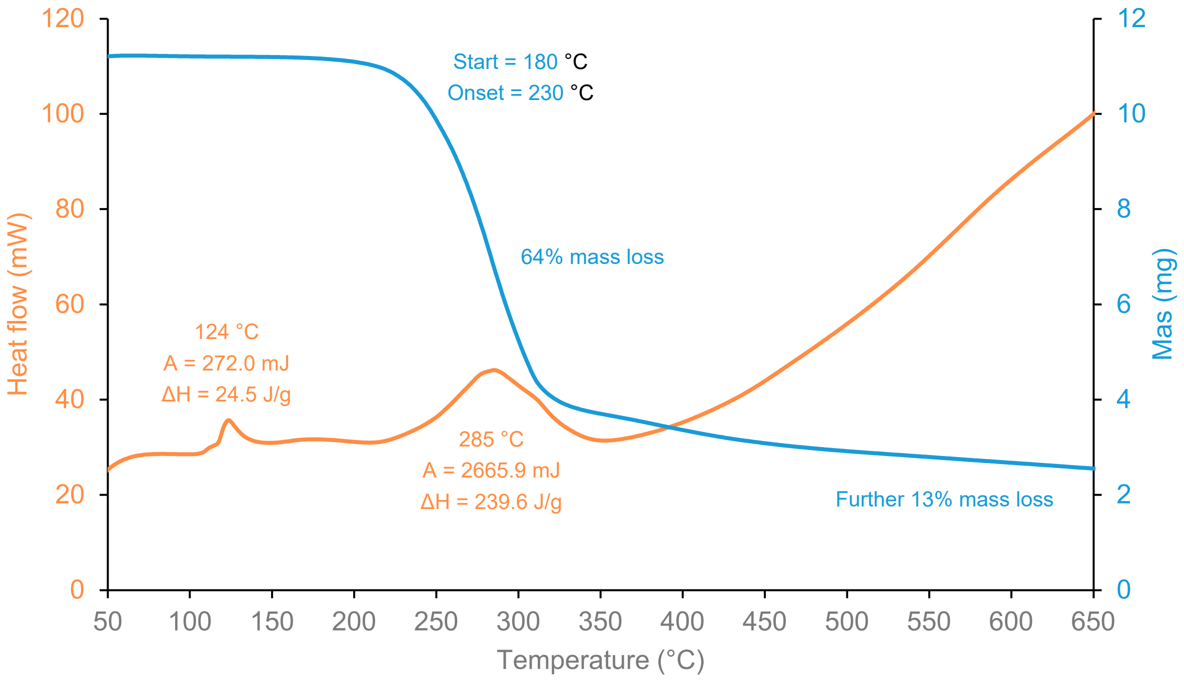

Figure S7a | STA analysis the polymer product of inverse vulcanisation of thraustochytrid oil. DSC trace in orange, TGA in blue. The exotherm at 124°C coincides with the melting point of sulfur and indicates the presence of unreacted S_8 within the polymer. Based on a calibration curve of sulfur melting events generated on the same instrument under the same conditions and published in *Laying Waste to Mercury: Inexpensive Sorbents Made from Sulfur and Recycled Cooking Oils, Supporting Information*, this exotherm indicates 51.1% of the mass of the polymer is free sulfur.

Unreacted sulfur constitutes a roughly half of the mass of the resultant polymer.

Dynamic Scanning Calorimetry for Glass Transition Temperature
Thraustochytrid oil Polymer

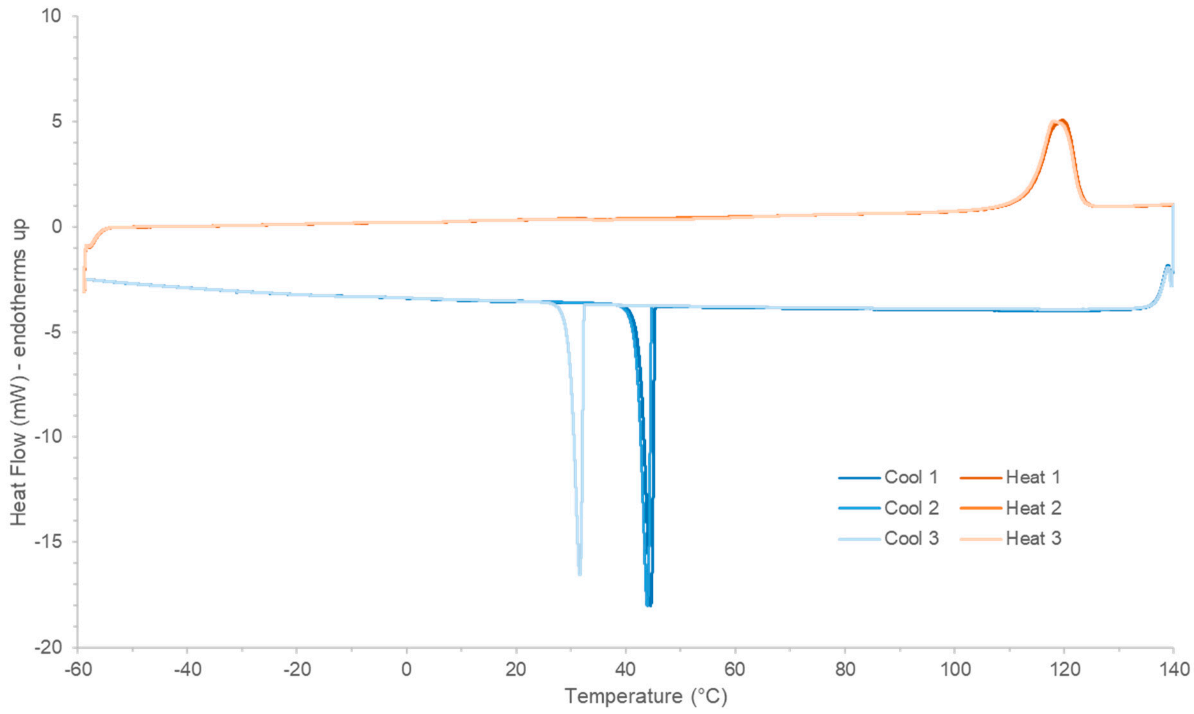

Figure S7b | DSC trace of thraustochytrid oil polymer

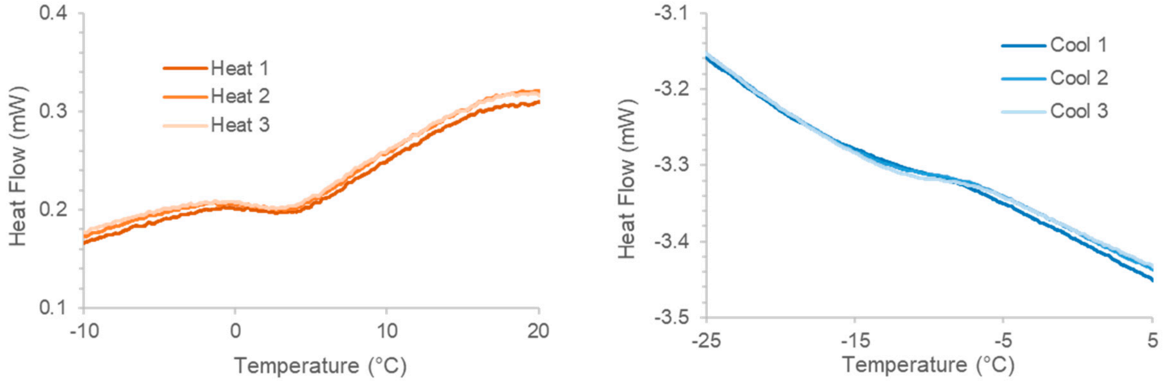

Figure S7c | DSC trace of thraustochytrid oil polymer, focused only on the baseline shift in the cooling and heating phases characteristic of a glass transition.

Heating Tg average = 1.1 °C (0.84, 0.98, 1.44)

Cooling Tg average = 11.4 °C (-12.41, -11.67, -10.26)

Combined average = **6.3 °C**

NMR Spectroscopy

Thraustochytrid oil Polymer

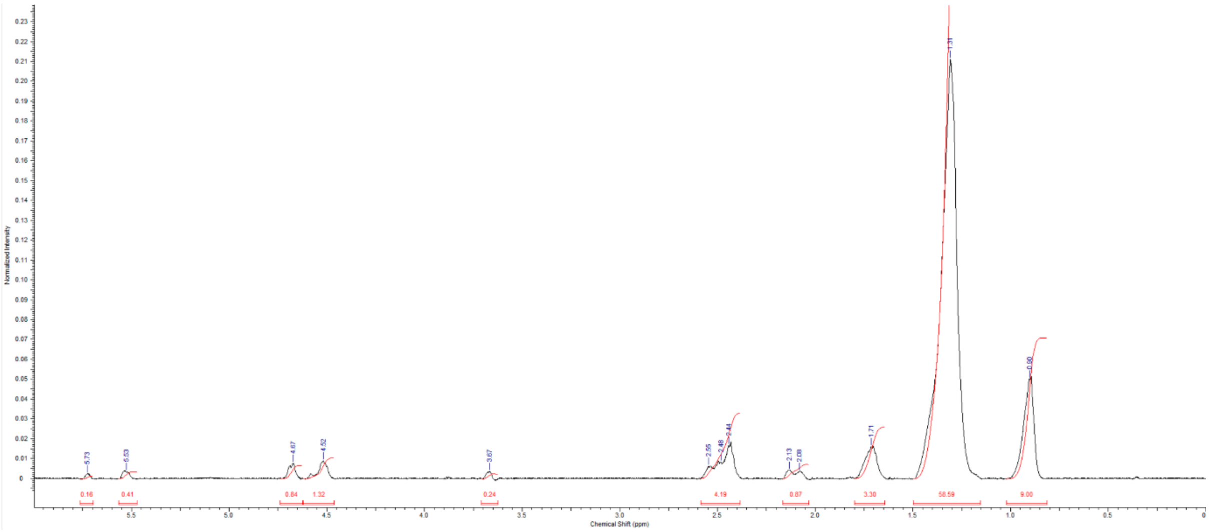

Figure S7d | ^{1}H NMR spectrum of thraustochytrid oil polymer in pyridine-d5. The spectrum indicates an average of 0.08 alkenes (shown as 0.16 alkene protons, δ = 5.73) per triglyceride molecule. Methyl end-group protons were used as a reference (9 per molecule, δ = 0.90 ppm).

X-ray Diffraction

Thraustochytrid oil Polymer

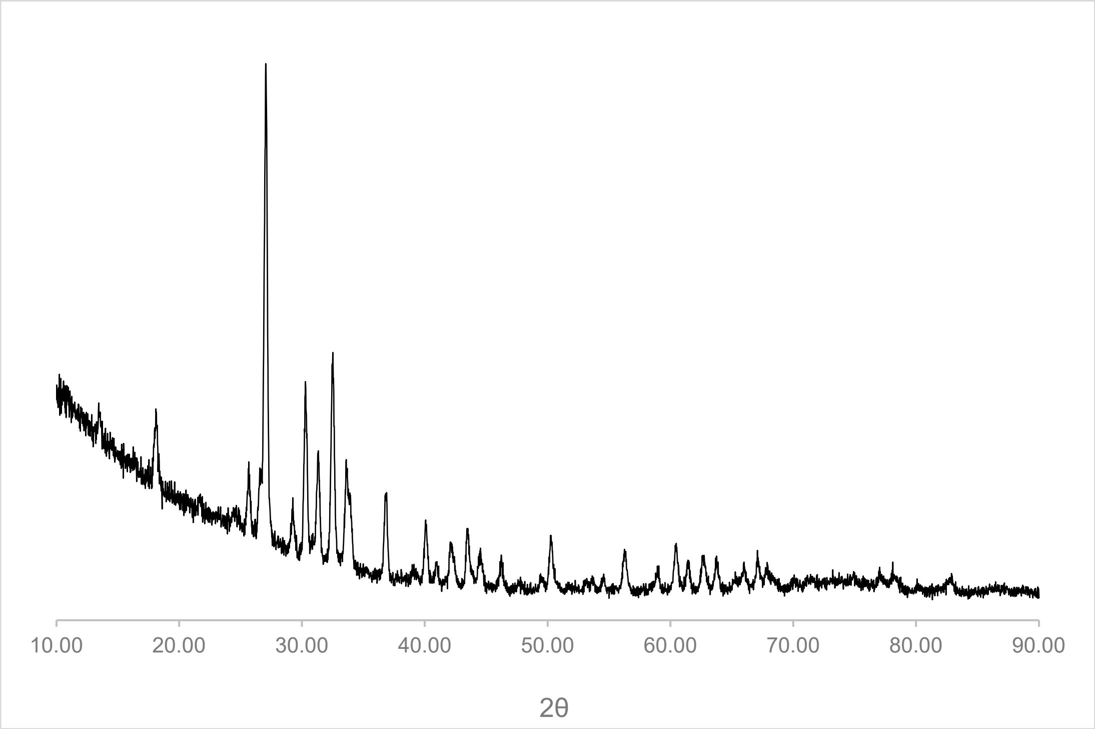

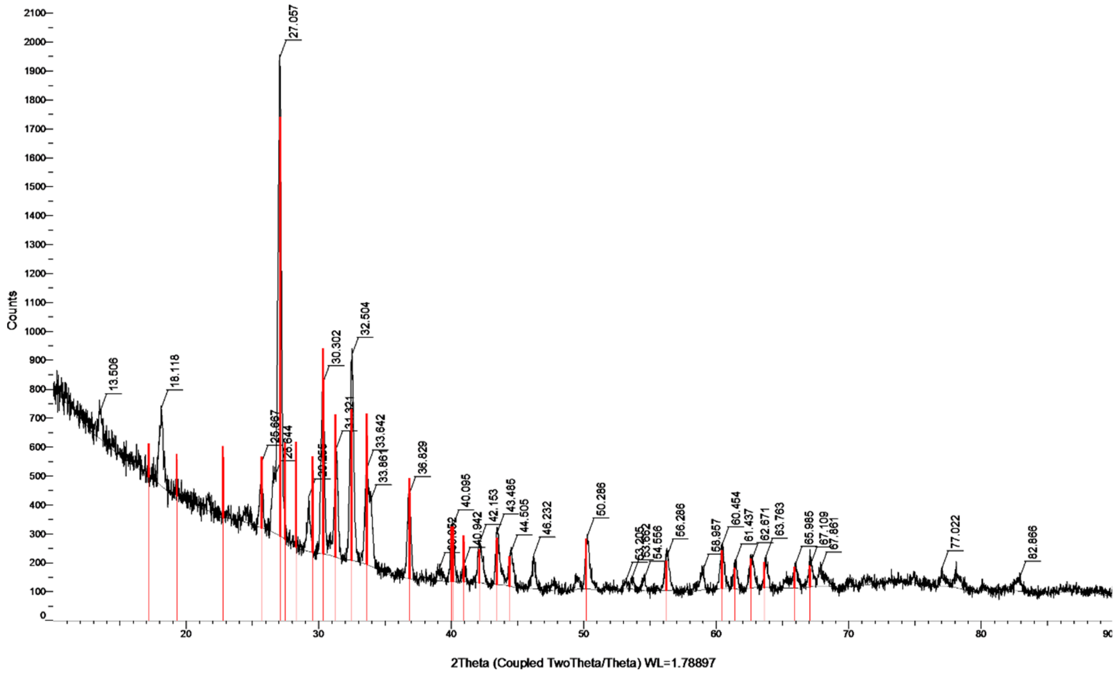

Figure S8 | Above: XRD spectrum of thraustochytrid oil polymer Below: sulfur (S_8) reference spectra overlayed in red.
